# Atrial cardiomyopathy markers predict ischemic cerebrovascular events independent of atrial fibrillation in patients with acute myocardial infarction

**DOI:** 10.3389/fcvm.2022.1025842

**Published:** 2022-11-22

**Authors:** Zhitong Li, Xin Wang, Quanbo Liu, Chenglin Li, Jinghan Gao, Yiheng Yang, Binhao Wang, Tesfaldet H. Hidru, Fei Liu, Xiaolei Yang, Yunlong Xia

**Affiliations:** ^1^Department of Cardiology, The First Affiliated Hospital of Dalian Medical University, Dalian, China; ^2^Department of Ultrasound, The First Affiliated Hospital of Dalian Medical University, Dalian, China; ^3^Department of Respiratory Medicine, Shengjing Hospital of China Medical University, Shenyang, China

**Keywords:** atrial cardiomyopathy, ischemic cerebrovascular events, P wave terminal force, left atrium diameter, B-type natriuretic peptide

## Abstract

**Background:**

Contemporary data on atrial cardiomyopathy (ACM) markers and ischemic cerebrovascular events (ICVE) in patients with acute myocardial infarction (AMI) is lacking. We aimed to examine whether ACM markers predict ICVE among AMI patients.

**Materials and methods:**

A total of 4,206 AMI cases diagnosed in clinical examinations between January 2016 and June 2021 were assessed for markers of ACM including B-type natriuretic peptide (BNP), P-wave terminal force in ECG lead V1 (PTFV1), and left atrium diameter (LAD). Left atrial enlargement (LAE) and abnormal PTFV1 were defined by previously published cut-off points. The primary outcome was incident ICVE composed of ischemic stroke (IS) and transient ischemic attack (TIA). Receiver operating curve analyses were used to compare the predictive performance of the CHA_2_DS_2_-VASc score combined with ACM markers to the CHA_2_DS_2_-VASc score alone.

**Results:**

During a median follow-up of 44.0 months, 229 (5.44%) ICVE occurred. Of these, 156 individuals developed IS and the remaining 73 cases were diagnosed with TIAs. The ICVE group showed larger PTFV1 and increased LAD as well as elevated BNP levels at baseline. In the multivariate analysis, we found significant associations with ICVE for PTFV1 (HR per 1,000 μV*ms, 1.143; 95% CI, 1.093–1.196), LAD (HR per millimeter, 1.148; 95% CI, 1.107–1.190), but not BNP after adjusting for known ICVE risk factors and interim atrial fibrillation (AF). The addition of abnormal PTFV1 and LAE improved the predictive accuracy of the CHA_2_DS_2_-VASc score with C-statistic increasing from 0.708 to 0.761 (*p* < 0.001).

**Conclusion:**

Atrial cardiomyopathy markers including PTFV1 and LAD were associated with incident ICVE independent of well-established risk factors and AF occurrence. The addition of ACM markers with CHA_2_DS_2_-VASc score may well discriminate individuals at high risk of ICVE in AMI patients.

## Introduction

Ischemic cerebrovascular event (ICVE) is one of the most dangerous complications after acute myocardial infarction (AMI), which is a well-established factor of poor prognosis (1). Previous studies have reported that new-onset atrial fibrillation (NOAF) is independently associated with ischemic stroke (IS) in acute coronary syndrome (ACS) patients (2). In contrast, some studies found that atrial cardiomyopathy (ACM) could cause cardioembolic stroke in the absence of AF (3). Whether NOAF is etiologically involved in the disease process or just a marker of ACM in patients with AMI remains unclear.

Over the recent years, increasing number of studies have significantly drawn attention to ACM, the complex disturbance in electrophysiology of the heart, or structural changes that negatively impact the normal function of the atria (4). According to a study conducted among the general population, ACM is considered to exist prior to AF and stroke (3). Although the diagnostic criteria for ACM are not clear at present, different biomarkers have been used to identify ACM (5, 6). In an ongoing cohort study, ACM is defined as NT-proBNP > 250 pg/mL, or P-wave terminal force in ECG lead V1 (PTFV1) > 5,000 μV*ms, or severe LAE (5).

The CHA_2_DS_2_-VASc score has been routinely used to assess future ICVE risk and guide anticoagulant therapy for patients with atrial fibrillation (AF) clinically. In recent years, the use of the CHA_2_DS_2_-VASc score in predicting ICVE has extended beyond the originally proposed. For instance, a recent study by Mitchell L. B. et al. reported that the CHA_2_DS_2_-VASc scores obtained similar ICVE predicting accuracy in patients with ACS but free of AF to that observed in populations with non-valvular AF (7). To the extent of our knowledge, no study investigated the association of ACM markers with ICVE and whether ACM markers could improve CHA_2_DS_2_-VASc scores to detect ICVE occurrence in AMI patients independent of AF. Therefore, the present study aimed to examine (a) the association between baseline ACM markers and ICVE occurrence, and (b) whether the addition of these markers to the CHA_2_DS_2_-VASc score would improve the prediction of ICVE in patients with AMI.

## Materials and methods

### Study participants

**FIGURE 1:**
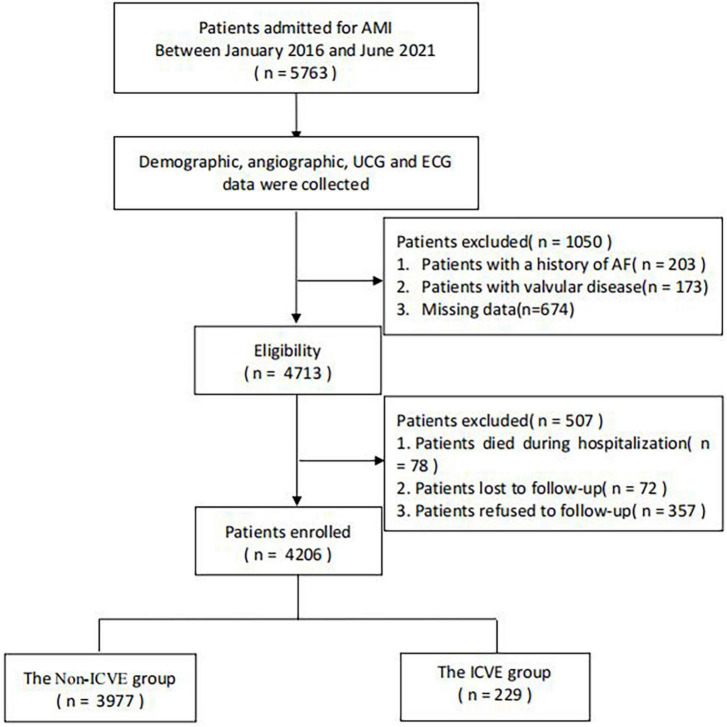
The overview of the selection of study participants. AF, atrial fibrillation; AMI, acute myocardial infarction; ECG, electrocardiogram; ICVE, ischemic cerebrovascular events; UCG, ultrasound cardiogram.

This hospital-based retrospective analysis was conducted among 5,763 AMI patients with complete clinical examinations and data on coronary angiography (CAG) between January 2016 and June 2021. Patients who died during hospitalization, patients with AF and valvular disease history, and patients who refused or were lost to follow-up were excluded. Ultimately, 4,206 patients were finally enrolled in this study. The remaining patients were categorized into two groups according to the presence of ICVE. The flow chart that demonstrates the included and excluded population is indicated in Figure 1. The Institutional Review Board of the First Affiliated Hospital of Dalian Medical University (FAHDMU) approved the study. This research abided and conform to the Helsinki declaration. The requirement for informed consent was waived due to the nature of our study design and all procedures comply with the approved research guidelines.

### Electrocardiogram parameters

The electrocardiogram (ECG) records were based on the initial ECG which was performed during AMI diagnosis. GE Healthcare MAC 5500 was used to record and download ECG parameters, which were calibrated at a speed of 25 mm/s with a voltage of 10 mm/1 mV. The multiplication of the duration (ms) and the depth (μV) of the terminal negative part the P wave in lead V1 was considered as PTFV1. In this study, we defined PTFV1 > 5,000 μV*ms as abnormal PTFV1 (5, 8). Of note, two independent cardiologists examined the PTFV1 values and their intra-observer correlation coefficient was found to be 0.92 (*P* < 0.001).

### Measurements and covariates

The electronic medical record of FAHDMU was searched for demographic, clinical, and laboratory data. An increase in systolic blood pressure (SBP) ≥ 140 mmHg or diastolic blood pressure (DBP) ≥ 90 mmHg or a history of antihypertensive drug use was defined as hypertension (HTN) (9). Diabetes mellitus (DM) was defined as previously [random blood sugar (RBS) level ≥ 200 mg/dL, fasting blood sugar (FBS) level ≥ 126 mg/dL or anti-diabetic drug use] (10). We defined dyslipidemia based on at least one of the below listed criteria: the presence of triglyceride (TG) ≥ 2.26 mmol/L (200 mg/dL), low density lipoprotein cholesterol (LDL-C) ≥ 4.14 mmol/L (160 mg/dL), high density lipoprotein cholesterol (HDL-C) ≤ 1.04 mmol/L (40 mg/dL), total cholesterol (TC) ≥ 6.22 mmol/L (240 mg/dL), or use of lipid-lowering medication (11). As previously defined in other studies, we compiled data from the clinical symptoms, echocardiography, chest X-ray, and electrocardiography to define congestive heart failure (CHF) (12). AF was defined based on 12-lead ECG or Holter ECG recordings. AMI was defined based on elevated cardiac troponin values (suggestive of myocardial injury) followed by one of the following criteria: (1) symptoms of myocardial ischemia, (2) ischaemic ECG changes, (3) evidence of pathological Q wave on ECG, or (4) availability of new regional wall motion abnormality in echocardiography. Further AMI was classified into ST-elevation myocardial infarction (STEMI) and non-ST-elevation myocardial infarction (NSTEMI) in accordance with the fourth universal definition of myocardial infarction (13).

### Follow-up

We obtained follow-up data either by reviewing medical records or by telephone interview. To obtain and censor ICVE outcomes, AMI patients were followed from their first admission until the occurrence of the primary outcome, death, or last follow-up (1 January 2022), whichever came first.

### Outcome assessment

The primary outcome was ICVE which was defined as fatal or non-fatal transient ischemic attack (TIA) or IS. In this study, IS was defined as new onset of a documented focal neurologic deficit lasting at least 24 h or until death or evidence of lesion on brain imaging. TIA was defined as a transient episode of focal neurologic deficit lasting less than 24 h and without brain imaging suggesting cerebral infarction.

### Statistical analysis

Continuous data were compared using the student’s *t*-test and the Mann–Whitney test depending on the nature of their distribution. The outcome was expressed as mean and standard deviation (SD) for normally distributed data and the median and interquartile range (IQR) for the non-normally distributed data. Categorical data were presented as count and percentage and differences were checked using the Chi-square test or Fisher exact test. Multivariate Cox proportional hazards models with incremental adjustments were used to examine the association of ACM markers with incident ICVE. Model 1 adjusted for age and gender. Model 2 included covariates from Model 1 plus variables with a *P*-value of < 0.05 in the univariate COX analyses and known variables associated with ICVE including prior history of hypertension, DM, stroke, peripheral arterial disease, CHF, SBP, heart rate at admission, KILLIP > 1, estimated glomerular filtration rate, log-transformed BNP, PTFV1 (per 1,000 μV*ms), left atrium diameter, left ventricular ejection fraction, use of diuretics, smoking status, and dyslipidemia. Model 3 included Model 2 covariates plus incident AF. We further dichotomized continuous ACM markers by the previously published cut-off points: abnormal PTFV1 (PTFV1 > 5,000 μV*ms) (5) and left atrial enlargement (LAE) (LAD > 38 mm for women and > 40 mm for men) (14). Subgroup analysis was executed between normal PTFV1 and abnormal PTFV1 groups as well as for normal LAD and LAE groups. The interaction between ACM markers and covariates was assessed with a Cox regression model.

The Kaplan–Meier curves and log-rank test were used to compare the freedom distributions and study the differences in ICVE freedom as stratified by ACM markers, respectively. We further executed time-dependent receiver operating characteristics of four different models, including CHA_2_DS_2_-VASc score (Model 1), CHA_2_DS_2_-VASc score + abnormal PTFV1 (Model 2), CHA_2_DS_2_-VASc score + LAE (Model 3), and CHA_2_DS_2_-VASc score + LAE + abnormal PTFV1 (Model 4). Harrell’s concordance statistics, a goodness of fit measure for models which produce risk scores, was calculated to measure the predictive power of ACM indicators and the combined models. The net reclassification index (NRI) was calculated to estimate the net change in the proportion of AMI patients assigned a more appropriate ICVE risk under the new model. Also, integrated discrimination improvement (IDI) was calculated to compare the discriminatory capacity among the models. *P*-value < 0.05 was considered statistically significant. All analyses were performed using R software.

## Results

### Baseline characteristics of the participants

A total of 4,206 patients (3,235 men and 971 women) were enrolled in the final analysis. After a median follow-up of 44.0 months, 229 individuals (5.44%) experienced incident ICVE (156 ISs and 73 TIAs). ICVE cases were older and likely to have more comorbidities such as hypertension, DM, stroke, peripheral arterial disease, and CHF. In addition, the ICVE group had higher values of log-transformed B-type natriuretic peptide (BNP), hypersensitive troponin I (hsTNI), PTFV1, left atrium diameter (LAD), and CHA_2_DS_2_-VASc score than those without ICVE. The Killip classification and the GRACE score were also higher in ICVE than in the non-ICVE group. Compared with non-ICVE cases, AMI patients with ICVE exhibited longer hospitalization lengths and more frequent in-hospital cardiac arrest (IHCA). At discharge, patients with ICVE were more likely to be prescribed diuretics than patients without ICVE. Participants in the ICVE group developed a higher proportion of AF (18.8 vs. 7.2%, respectively) during the follow-up than those in ICVE free group. The demographic and baseline clinical characteristics of the AMI patients included in the analysis are shown in Table 1.

**TABLE 1:** Baseline characteristics.

Variable	Overall	No ICVE	ICVE	*P*-value
	(*n* = 4206)	(*n* = 3977)	(*n* = 229)	
Age, years	62.9 (11.9)	62.6 (11.9)	67.6 (10.6)	< 0.001
Male, *n* (%)	3235 (76.9)	3060 (76.9)	175 (76.4)	0.855
Smoking, *n* (%)	1940 (46.1)	1843 (46.3)	97 (42.4)	0.240
Drinking, *n* (%)	897 (21.3)	853 (21.4)	44 (19.2)	0.472
**Medical history**				
HTN, *n* (%)	2456 (58.4)	2293 (57.7)	16 (71.2)	< 0.001
DM, *n* (%)	1450 (34.5)	1343 (33.8)	107 (46.7)	< 0.001
Dyslipidemia, *n* (%)	2790 (66.3)	2634 (66.2)	156 (68.1)	0.605
Prior MI, *n* (%)	54 (1.3)	52 (1.3)	2 (0.9)	0.791
Previous stroke, *n* (%)	278 (6.6)	226 (5.7)	52 (22.7)	< 0.001
PAD, *n* (%)	413 (9.8)	374 (9.4)	39 (17.0)	< 0.001
CHF, *n* (%)	272 (6.5)	241 (6.1)	31 (13.5)	< 0.001
**Initial presentation**				
SBP, mmHg	131.2 (24.3)	130.9 (24.2)	136.3 (24.8)	0.001
DBP, mmHg	78.1 (13.4)	78.1 (13.4)	79.3 (12.9)	0.169
HR at admission, b.p.m.	74.8 (15.6)	74.7 (15.6)	77.3 (15.7)	0.014
KILLIP > 1, *n* (%)	683 (16.2)	627 (15.8)	56 (24.5)	0.001
STEMI, *n* (%)	2004 (47.6)	1903 (47.9)	101 (44.1)	0.300
Anterior wall, *n* (%)	969 (48.4)	919 (48.3)	50 (49.5)	0.812
Inferior wall, *n* (%)	1026 (51.2)	978 (51.4)	48 (47.5)	0.449
Others, *n* (%)	392 (19.6)	370 (19.4)	22 (21.8)	0.564
CHA_2_DS_2_-VASc Score	2 (1–3)	2 (1–3)	3 (2–4)	< 0.001
GRACE score	143.0 (31.8)	142.4 (31.8)	152.8 (31.0)	< 0.001
**Culprit lesion**				
LM, *n* (%)	93 (2.2)	89 (2.2)	4 (1.7)	0.795
LAD, *n* (%)	1669 (39.7)	1573 (39.6)	96 (41.9)	0.520
LCX, *n* (%)	717 (17.0)	678 (17.0)	39 (17.0)	1.000
RCA, *n* (%)	1402 (33.3)	1321 (33.2)	81 (35.4)	0.548
**Laboratory data and ECG parameters**				
eGFR, ml/(min⋅1.73 m^2^)	91.2 (28.3)	91.8 (28.1)	80.6 (30.8)	< 0.001
Uric Acid, μmol/L	353 (291–415)	353 (292–414)	361 (286–448)	0.220
BNP, pg/ml	124 (52–311)	122 (51–294)	255 (84–578)	< 0.001
LogBNP	7.0 (5.7–8.3)	6.9 (5.7–8.2)	8.0 (6.4–9.2)	< 0.001
hsTnI, pg/ml	8.4 (1.2–54.4)	8.8 (1.2–56.0)	5.0 (0.7–25.7)	0.003
PTFV1, μV*ms	2210 (0–3710)	2112 (0–3552)	3944 (2279–5655)	< 0.001
**Echocardiographic parameters**				
LAD, mm	37.4 (3.7)	37.2 (3.6)	39.9 (4.7)	< 0.001
LVEF, %	51.8 (8.4)	51.9 (8.4)	50.4 (9.5)	0.023
**Initial treatment**				
PCI, *n* (%)	3692 (87.8)	3490 (87.8)	202 (88.2)	0.920
CABG, *n* (%)	35 (0.8)	33 (0.8)	2 (0.9)	1.000
Thrombolysis, *n* (%)	50 (1.2)	49 (1.2)	1 (0.4)	0.443
Length of hospitalization, day	6 (5–7)	6 (5–7)	6 (5–8)	0.001
IHCA, *n* (%)	88 (2.1)	78 (2.0)	10 (4.4)	0.025
Incident AF, *n* (%)	331 (7.9)	288 (7.2)	43 (18.8)	< 0.001
**Medication at discharge**				
ACEI/ARB, *n* (%)	2900 (68.9)	2731 (68.7)	169 (73.8)	0.119
βblocker, *n* (%)	3373 (80.2)	3180 (80.0)	193 (84.3)	0.131
Statins, *n* (%)	4190 (99.6)	3962 (99.6)	228 (99.6)	1.000
OAC, *n* (%)	62 (1.5)	56 (1.4)	6 (2.6)	0.231
Aspirin, *n* (%)	4156 (98.8)	3931 (98.8)	225 (98.3)	0.626
P2Y_12_ receptor inhibitor, *n* (%)	4200 (99.9)	3971 (99.8)	229 (100.0)	1.000
Diuretic, *n* (%)	1097 (26.1)	1015 (25.5)	82 (35.8)	0.001

ACEI, angiotensin-Converting Enzyme Inhibitors; ARB, angiotensin-converting enzyme receptor blockers; BNP, B-type natriuretic peptide; CABG, coronary artery bypass grafting; CHF, congestive heart failure; DM, diabetes mellitus; eGFR, estimated glomerular filtration rate; GRACE, global registry of acute coronary events; HR, heart rate; hsTnI, hypersensitive troponin I; HTN, hypertension; IHCA, in-hospital cardiac arrest; LAD, left anterior descending coronary artery; LAD, left atrium diameter; LCX, left coronary circumflexus artery; LM, left main coronary artery; LVEF, left ventricular ejection fraction; MI, myocardial infarction; OAC, oral anticoagulants; PAD, peripheral arterial disease; PCI, percutaneous coronary intervention; PTFV1, P-wave terminal force in ECG lead V1; RCA, right coronary artery; SBP, systolic blood pressure; STEMI, ST-elevation myocardial infarction.

### Relationship between atrial cardiomyopathy markers, incident atrial fibrillation and ischemic cerebrovascular events

**FIGURE 2:**
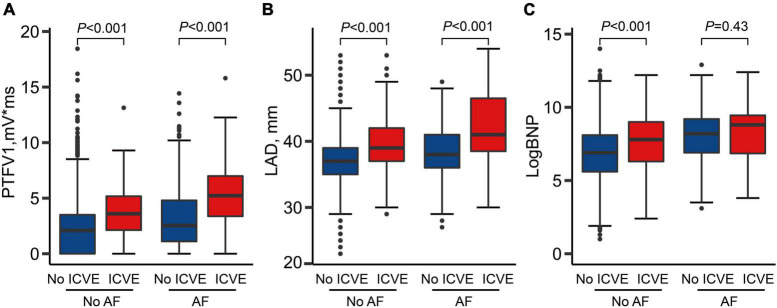
Atrial cardiomyopathy markers by strata of atrial fibrillation (AF) and ischemic cerebrovascular events (ICVE). **(A)** PTFV1 levels by strata of AF and ICVE; **(B)** LAD levels by strata of AF and ICVE; **(C)** LogBNP levels by strata of AF and ICVE.

Figure 2 illustrates the comparison of ACM markers between ICVE and non-ICVE groups with or without incident AF. Individuals with ICVE had larger LAD and PTFV1 values than those without ICVE regardless of their AF status (*P* < 0.001). Similarly, ICVE patients had higher LogBNP values than those without ICVE in non-AF patients (*P* < 0.001). However, there were no significant differences in LogBNP values between ICVE and non-ICVE groups in patients with incident AF (Figure 2).

Univariable analysis between baseline and incident ICVE for the entire cohort were shown in Supplementary Table 1. In the multivariate model (Model 2), we found positive relationship between PTFV1 and incident ICVE (HR per 1,000 μV*ms, 1.148; 95% CI, 1.097–1.201, *P* < 0.001), LAD (HR per millimeter, 1.152; 95% CI, 1.111–1.194, *P* < 0.001) but not for LogBNP (HR per doubling of BNP, 1.058; 95% CI, 0.962–1.164, *P* = 0.244).

To investigate the effect of AF, we considered incident AF as a single variable during the adjustment for the multivariate model. The association between ACM markers and ICVE occurrence was attenuated but still remained significant for PTFV1 (HR per 1,000 μV*ms, 1.140; 95% CI: 1.090–1.193, *P* < 0.001) and LAD (HR: 1.147 per millimeter; 95% CI: 1.106–1.189, *P* < 0.001), suggesting the association between ACM markers and ICVE independent of AF occurrence. The association between ACM markers and ICVE is shown in Table 2. To test whether the proportional hazard assumption was satisfied, we checked Schoenfeld residual tests. The result indicated that there was no collinearity violation between Schoenfeld residuals and time (Supplementary Figure 1).

**TABLE 2:** The relationship between ACM markers and ICVE.

	Model 1		Model 2		Model 3	
	Hazard ratio	*P*-value	Hazard ratio	*P*-value	Hazard ratio	*P*-value
PTFV1 (per 1000 μV*ms)	1.231 (1.182–1.281)	< 0.001	1.148 (1.097–1.201)	< 0.001	1.140 (1.090–1.193)	< 0.001
LAD, mm	1.199 (1.161–1.238)	< 0.001	1.152 (1.111–1.194)	< 0.001	1.147 (1.106–1.189)	< 0.001
LogBNP	1.279 (1.183–1.383)	< 0.001	1.058 (0.962–1.164)	0.244	1.053 (0.957–1.159)	0.288

Model 1 adjusted for age and gender. Model 2 included covariates from model 1 plus smoking status, prior history of hypertension, diabetes mellitus, dyslipidemia, stroke, peripheral arterial disease, congestive heart failure, systolic blood pressure, heart rate at admission, KILLIP > 1, estimated glomerular filtration rate, log-transformed BNP, PTFV1 (per1000 μV*ms), left atrium diameter, left ventricular ejection fraction and use of diuretics. Model 3 included Model 2 covariates plus incident AF.

### Subgroup analysis

**FIGURE 3:**
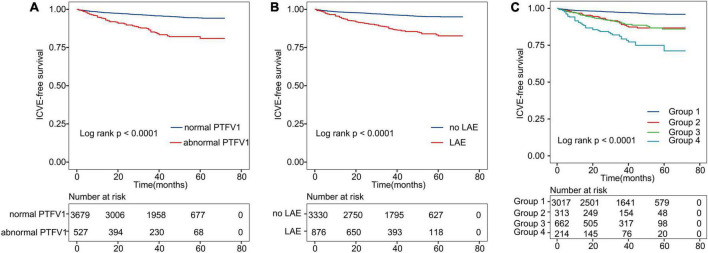
Kaplan–Meier curves show ICVE-free survival. **(A)** Kaplan–Meier survival curves for abnormal PTFV1. **(B)** Kaplan–Meier survival curves for LAE. **(C)** All patients were divided into four categories. The lines represent the following: Group 1: patients with both normal PTFV1 and no LAE; Group 2: patients with abnormal PTFV1 and no LAE; Group 3: patients with LAE and normal PTFV1; Group 4: patients with both abnormal PTFV1 and LAE. ICVE, ischemic cerebrovascular events.

We further dichotomized continuous PTFV1 and LAD covariates based on previously published cut-off points to run a sub-group analysis. Supplementary Figures 2, 3 present the prognostic effect of PTFV1 and LAD in different subgroups. We observed a low risk of ICVE in the normal PTFV1 and LAD groups. However, our data indicate that there were obvious differences in ICVE occurrence between patients with normal and abnormal PTFV1 (Figure 3A). Similarly, we observed a significant difference in ICVE occurrence between normal LAD and LAE groups (Figure 3B). Hence, the applied cut-off points can effectively draw the line for the ICVE risk between the lower-risk and higher-risk groups.

Further, we divided patients into four groups based on the previously defined cut-off points of PTFV1/LAD (Figure 3C). Each group represented combinations of two different markers: Group 1: patients with both normal PTFV1 and LAD; Group 2: patients with abnormal PTFV1 and normal LAD; Group 3: patients with LAE and normal PTFV1; Group 4: patients with abnormal PTFV1 and LAE. Patients in group 4 had the highest incidence of ICVE (log-rank test, *P* < 0.001).

### The combined effect of atrial cardiomyopathy markers on the CHA_2_DS_2_-VASc score

**FIGURE 4:**
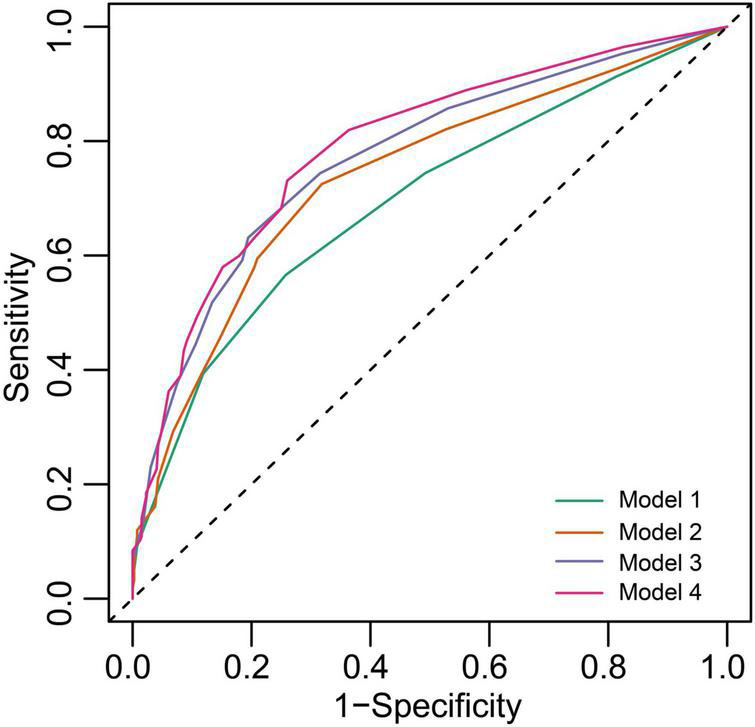
Receiver operating characteristics (ROC) curves of freedom from ICVE at 5 years for the different risk prediction models. Model 1, CHA_2_DS_2_-VASc score; Model 2, CHA_2_DS_2_-VASc score + abnormal PTFV1; Model 3, CHA_2_DS_2_-VASc score + LAE; Model 4, CHA_2_DS_2_-VASc score + abnormal PTFV1 + LAE.

The results of the receiver operating characteristics (ROC) analysis that compared the performance of ACM markers (PTFV1 and LAD) against the CHA_2_DS_2_-VASc score to discriminate the ICVE patients are indicated in Figure 4. The CHA_2_DS_2_-VASc score alone had a moderate predictive ability, with a C-Statistic of 0.708 (95% CI: 0.667–0.749). The C-Statistic of the CHA_2_DS_2_-VASc score + abnormal PTFV1 and CHA_2_DS_2_-VASc score + LAE were 0.743 (95% CI: 0.707–0.779) and 0.742 (95% CI: 0.708–0.776), respectively. Notably, the greatest improvement in CHA_2_DS_2_-VASc predictive utility was observed when both abnormal PTFV1 and LAE were added, with C-Statistic increasing from 0.708 to 0.761 (*P* < 0.001) (Table 3). The IDI and NRI output demonstrates the superiority of the combined model compared to the CHA_2_DS_2_-VASc score alone, suggesting that the use of the combined final model could stratify the risk of ICVE better than the CHA_2_DS_2_-VASc score alone.

**TABLE 3:** Comparison of different risk prediction models.

	Model 1	Model 2	Model 3	Model 4
AUC (95% CI)	0.700 (0.657–0.743)	0.742 (0.701–0.783)	0.757 (0.719–0.796)	0.782 (0.745–0.819)
*P*-value	–	0.002	< 0.001	< 0.001
C-Statistic (95% CI)	0.708 (0.667–0.749)	0.743 (0.707–0.779)	0.742 (0.708–0.776)	0.761 (0.729–0.793)
*P*-value	–	< 0.001	0.002	< 0.001
IDI (95% CI)	Ref	0.022 (0.010–0.041)	0.027 (0.013–0.045)	0.041 (0.025–0.067)
*P*-value	–	< 0.001	< 0.001	< 0.001
NRI (95% CI)	Ref	0.211 (0.149–0.271)	0.309 (0.230–0.383)	0.384 (0.308–0.457)
*P*-value	–	< 0.001	< 0.001	< 0.001

Model 1, CHA_2_DS_2_-VASc score; Model 2, CHA_2_DS_2_-VASc score + abnormal PTFV1; Model 3, CHA_2_DS_2_-VASc score + LAE; Model 4, CHA_2_DS_2_-VASc score + abnormal PTFV1 + LAE. AUC, Area under ROC curve; IDI, integrated discrimination improvement; NRI, net reclassification index.

## Discussion

The findings of the present study demonstrated that two ACM markers including abnormal PTFV1 and LAD were positively linked with a substantial risk of incident ICVE in the Chinese population with AMI. The relationship persisted even after adjusting for conventional cerebrovascular disease risk factors and interim incident AF. The addition of abnormal PTFV1 and LAE to the CHA_2_DS_2_-VASc score significantly improved the prediction of ICVE risk.

The CHA_2_DS_2_-VASc score has been used to assess the individual stroke risk and determine anticoagulation therapy indications for AF patients in routine clinical practice. Current guidelines recommend short-term use of triple antithrombotic treatment including dual antiplatelet therapy (DAPT) and oral anticoagulants in high-risk individuals (CHA_2_DS_2_-VASc score ≥ 2) with AMI and AF for stroke prevention (15). However, ICVE could even occur in the absence of AF (16). This indicates intensive work is needed to efficiently identify high-risk patients and to improve the currently available risk stratification approaches. In the present study, we found that LAE and abnormally increased PTFV1 improved the predictive ability of the CHA_2_DS_2_-VASc score for ICVE. These findings suggest that the addition of LAE and abnormal PTFV1 with a CHA_2_DS_2_-VASc score may offer an improved predictive capacity performance for ICVE in individuals with AMI.

Recent evidence showed that ACM summarizes pathological functional, electrical, and structural remodeling in the atria (4) and could result in a pro-arrhythmogenic and pro-thrombotic atrial substrate. Several prospective studies with large sample sizes showed that ACM markers (including PTFV1, LAD, and NT-proBNP/BNP) could predict the occurrence of ICVE (3) in the general population. To our knowledge, no study has reported the predictive role of ACM markers for ICVE in AMI cases independent of AF. Hence, this study reflects the significant relationship between the ACM indicators (particularly, LAE and abnormal PTFV1) and ICVE after AMI, even after considering the confounding effect of several known risk factors and incident AF. This article built on previous literature which was conducted in general population, verifying and expanding the clinical applications of ACM markers to predict ICVE in patients with AMI. The main difference between the present study and previous studies is that BNP value was not associated with ICVE in AMI patients. Our results are in line with the previous studies (17). One possible reason for the lack of such association is that hemodynamics was not stable during AMI and BNP levels are vulnerable to hemodynamic changes. Therefore, the BNP level during the acute phase of myocardial infarction is not a good reflection of the long-term pressure overload after AMI. Atrial natriuretic peptide (ANP), a member of the natriuretic peptide hormone family, is released from the atria in response to stretch, as a result, elevated ANP level may reflect increased filling pressure and dysfunction of atria. ANP is reported to be associated with incidence of AF and stroke (18). Moreover, ANP significantly improved the prediction of AF and stroke when added to a predictive model consisting of conventional risk factors (19). Due to the retrospective nature of this study, serum ANP level was not available. Further studies should be performed to investigate the association between ANP level and ICVE in AMI patients and whether ANP could serve as ACM biomarkers in clinical practice.

The PTFV1 is a widely reported indicator for left atrial-related changes independent of structural deformity or pressure alterations in the left atrium of the heart (20, 21). Therefore, it has been considered a marker for electrical and functional remodeling of the atria (22). Besides, there exists substantial evidence regarding the link between PTFV1 and stroke (especially cryptogenic or cardioembolic stroke) regardless of AF in the general population (23). Abnormal PTFV1 may reflect atrial changes, such as fibrosis, delayed interatrial conduction, increased LA volume, and decrease LA function (24), all of which are reported to be associated with IS. The present study further corroborates the association between PTFV1 and ICVE among patients with AMI, even after adjustment for other indicators of ACM such as LAD and BNP, suggesting PTFV1 may reflect atrial changes that could not be fully represented by echocardiographic or serum biomarkers. Therefore, PTFV1, which is easily available in clinical practice and does not require complex calculations, can be beneficial as a cost-effective prognostic marker to recognize subjects at high risk for ICVE after AMI.

Tissue fibrosis and abnormally enlarged atrial size, which could spot by echocardiography, indicate the sign of left atrial remodeling (25). Previous studies showed that left atrial size was a significant risk factor for stroke or stroke recurrence, after adjustment for incident AF (26, 27). In addition, LAE was also shown to increase IS risk in patients with sinus rhythm across studies (28). We similarly found a positive relationship between LAE and ICVE after adjustment for incident AF and other risk factors of stroke. The underlying etiology behind the higher risk is likely to be multifactorial. It is also important to consider that LAE and ICVE share similar risk factors, namely, advanced age, hypertension, diastolic dysfunction, and left ventricular hypertrophy (29). In this study, the relationship between LAD and incident ICVE was diminished after adjusting for the aforementioned variables and other known stroke risk factors. This may imply that the mechanism of ICVE in patients with LAE could be partially explained by coexisting risk factors. In the past, AMI patients with LAE were found to develop new-onset AF (30). Apparently, reduced flow velocity in the left atrial appendage due to an increase in left atrial volume contributes to stasis and clot formation. This is consistent with transesophageal echocardiographic data which suggest that LAE was an independent risk factor for left atrial thrombus or spontaneous echocardiographic contrast and embolic events (31). Moreover, our study consolidated the relationship between the LAE and ICVE in AMI patients, and LAE may also represent a potential indication either for initiating or monitoring anticoagulant therapy for the prevention of stroke before the onset of AF in patients with AMI (17).

In the past, it was generally held that AF is a major cause of IS due to the blood flow stasis and thrombus formation in the LA during AF episodes. However, the lack of a clear temporal association between AF episodes and stroke development (16, 32) and the comparable risk of stroke between rate- and rhythm-control strategies among AF patients have challenged this theory. The above findings drive us to rethink the relationship between AF and stroke and a new model including both atrial substrate and the AF in thrombogenesis has been well established (33). In this model, AF is no longer necessary for stroke. An abnormal atrial substrate may cause thromboembolism independent of AF despite AF being associated with increased thromboembolic risk. This implies that AF was more likely a marker of later stages of ACM rather than an etiology of stroke. Our findings are similar to this model, as both abnormal PTFV1 and LAE, two markers of ACM, were associated with the development of ICVE independent of incident AF.

Additionally, the low usage rate of oral anticoagulation (OAC) in our population which was consistent with a Chinese national registry (34) might also have contributed to the increased risk of ICVE. According to previous studies, appropriate caution of OAC consideration is vital after AMI because the addition of OAC to DAPT does not significantly prevent thromboembolism (35, 36). Another study detected a decrease in stroke recurrence with rivaroxaban over aspirin therapy in individuals with LAE (37). Whether ACM markers could aid in identifying AMI patients who were most likely to benefit from anticoagulation in addition to antiplatelet therapies remain unclear. In the future, further multi-institutional prospective studies with a greater number of subjects are warranted to investigate the optimal antithrombotic therapy both assessing atrial rhythm and substrate to permit efficient anticoagulant therapy for high-risk patients while avoiding unnecessary bleeding events from anticoagulation for those at low risk.

### Study limitations

This was a retrospective study with inherent limitations. First, the number of patients who developed ICVE in this study is limited, further prospective, multicenter, large-sample studies are highly desirable. Second, data on stroke subtypes were unavailable. The likelihood between left atrial abnormality and ICVE may be partly due to atherosclerosis. In addition, due to the retrospective nature of the study, left atrial volume index and left atrial speckle tracking which are considered as more reliable parameters to represent left atrial structural and functional remodeling as well as other risk factors for ICVE were not available in the present study. More future studies adjusting more comprehensive confounding factors should be designed to investigate the relationship between these parameters and ICVE and whether ACM is an independent risk factor of ICVE in AMI patients in the future. Third, participants did not undergo continuous heart-rhythm monitoring to detect subclinical AF. As 80–90% of AF cases were asymptomatic (38), some patients with pre-existing asymptomatic AF may either remain undiagnosed or erroneously regarded to have NOAF. Finally, the prevalence of asymptomatic brain vascular lesions is substantially higher than the clinically overt disease (39). Participants did not undergo regular brain imaging examinations to rule out subclinical IS, and we therefore may underestimate the number of patients with ICVE.

## Conclusion

In this study, ACM markers including abnormal PTFV1 and LAE were independently associated with ICVE. The addition of abnormal PTFV1 and LAE could improve the ICVE risk prediction of the CHA_2_DS_2_-VASc risk score in patients with AMI. Further prospective studies are warranted to confirm these findings.

## Data availability statement

The raw data supporting the conclusions of this article will be made available by the authors, without undue reservation.

## Ethics statement

The studies involving human participants were reviewed and approved by the Institutional Review Board of First Affiliated Hospital of Dalian Medical University. Written informed consent for participation was not required for this study in accordance with the national legislation and the institutional requirements.

## Author contributions

XY and YX designed the study. ZL, XW, and QL were in charge of the data analysis. ZL drafted the article. JG, YY, and BW conducted the data collection. CL was in charge of the data administration and the literature collection. CL, XW, QL, TH, and FL did the critical revision of the article. All authors have read and approved the final manuscript.
