# Supplementary material for: Atrial cardiomyopathy markers predict ischemic cerebrovascular events independent of atrial fibrillation in patients with acute myocardial infarction
Source: Front Cardiovasc Med. 2022 Nov 22;9:1025842. doi: 10.3389/fcvm.2022.1025842 (PMC9722740; doi:10.3389/fcvm.2022.1025842)

**supplementary materials**

**Table S1.Univariate COX Regression Analysis of ICVE**

| Variable | Univariate analysis | |
| --- | --- | --- |
|  | HR(95%CI) | P value |
| Age, years | 1.040(1.028-1.053) | <0.001 |
| Male sex | 0.935(0.689-1.269) | 0.666 |
| Smoking | 0.814(0.626-1.058) | 0.123 |
| Drinking | 0.882(0.635-1.226) | 0.455 |
| **Medical history** |  |  |
| HTN | 1.863(1.399-2.480) | <0.001 |
| DM | 1.781(1.373-2.309) | <0.001 |
| Prior MI | 0.694(0.172-2.791) | 0.607 |
| Previous stroke | 5.242(3.845-7.146) | <0.001 |
| PAD | 2.087(1.478-2.946) | <0.001 |
| CHF | 2.806(1.920-4.100) | <0.001 |
| **Initial presentation** | |  |
| SBP,mmHg | 1.010(1.004-1.015) | <0.001 |
| DBP,mmHg | 1.008(0.998-1.017) | 0.121 |
| HR at admission, b.p.m. | 1.012(1.004-1.020) | 0.002 |
| KILLIP>1 | 1.862(1.377-2.517) | <0.001 |
| STEMI | 0.816(0.628-1.059) | 0.126 |
| **Culprit lesion** |  |  |
| LM | 0.875(0.326-2.351) | 0.791 |
| LAD | 1.091(0.839-1.419) | 0.515 |
| LCX | 0.992(0.703-1.401) | 0.965 |
| RCA | 1.069(0.816-1.402) | 0.627 |
| **laboratory data and ECG parameters** | |  |
| eGFR, ml/(min ▪1.73 m^2^) | 0.985(0.980-0.989) | <0.001 |
| Uric Acid,µmol/L | 1.001(1.000-1.002) | 0.055 |
| LogBNP | 1.388(1.244-1.438) | <0.001 |
| hsTnI,pg/mL | 0.999(0.997-1.000) | 0.077 |
| Dyslipidemia | 1.141(0.864-1.507) | 0.353 |
| PTFV1(per1000μV*ms) | 1.240(1.194-1.287) | <0.001 |
| **Echocardiographic parameters** | |  |
| LAD, mm | 1.197(1.161-1.234) | <0.001 |
| LVEF, % | 0.975(0.961-0.989) | 0.001 |
| **Initial treatment** |  |  |
| PCI | 0.994(0.665-1.486) | 0.978 |
| CABG | 1.084(0.269-4.359) | 0.910 |
| Thrombolysis | 0.303(0.043-2.163) | 0.234 |
| Incident AF | 3.078(2.209-4.289) | <0.001 |
| **Medication at discharge** |  |  |
| ACEI/ARB | 1.238(0.922-1.661) | 0.156 |
| βblocker | 1.318(0.924-1.882) | 0.128 |
| Statins | 0.870(0.122-6.205) | 0.890 |
| OAC | 2.116(0.940-4.763) | 0.070 |
| Aspirin | 0.581(0.216-1.563) | 0.282 |
| Diuretic | 1.675(1.278-2.195) | <0.001 |

Abbreviations: ACEI=angiotensin-Converting Enzyme Inhibitors; ARB=angiotensin-converting enzyme receptor blockers; BNP=brain natriuretic peptide;CABG=coronary artery bypass grafting; CHF=Congestive heart failure; DBP=diastolic blood pressure; DM=Diabetes Mellitus; eGFR=estimated glomerular filtration rate; HR=heart rate; hsTnI=hypersensitive troponin I; HTN=Hypertension; LAD=left atrium diameter; LAD=left anterior descending coronary artery; LCX=left coronary circumflexus artery; LVEF= left ventricular ejection fraction; MI=myocardial infarction; OAC=oral anticoagulants; PAD=peripheral arterial disease; PCI=percutaneous coronary intervention; RCA=right coronary artery; PTFV1=P-wave terminal force in ECG lead V1; SBP=systolic blood pressure; STEMI=ST-elevation myocardial infarction;

### Figure Legends

FigS1 Analysis of the residuals of Schoenfeld residuals to assess the proportionality assumption. (A) plots of beta-coefficient estimates (log hazard ratios) for PTFV1 against follow-up (time) in months. (B) plots of beta-coefficient estimates (log hazard ratios) for LAD against follow-up (time) in months. (C) plots of beta-coefficient estimates (log hazard ratios) for LogBNP against follow-up (time) in months. The black solid line represents a smoothed curve of scaled Schoenfeld residuals with 95% confidence intervals (black dotted lines).

FigS2. Subgroup analysis for ICVE. The effect of abnormal PTFV1 on the risk of ICVE was consistent across various subgroups. CI =confidence interval; DM=Diabetes Mellitus; HBP=Hypertension; LAE= left atrial enlargement; LVEF= left ventricular ejection fraction; NSTEMI=Non-ST-elevation myocardial infarction; STEMI=ST-elevation myocardial infarction;

FigS3. Subgroup analysis for ICVE. The effect of LAE on the risk of ICVE was consistent across various subgroups. CI =confidence interval; DM=Diabetes Mellitus; HBP=Hypertension; LVEF= left ventricular ejection fraction; NSTEMI=Non-ST-elevation myocardial infarction; STEMI=ST-elevation myocardial infarction;

**FigS1**


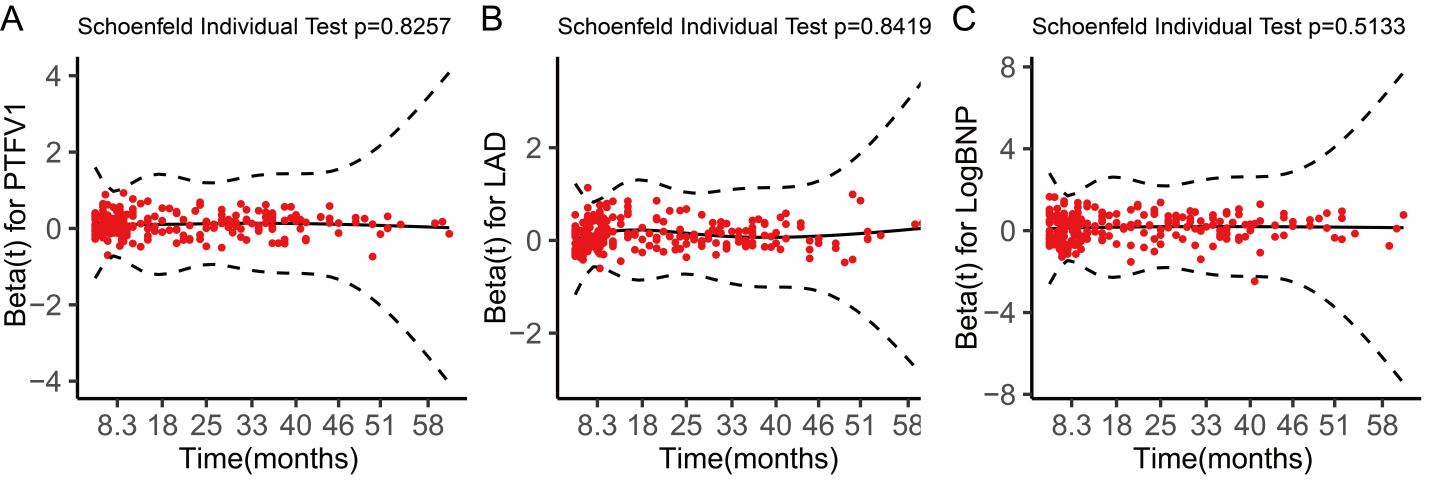


**FigS2**

**
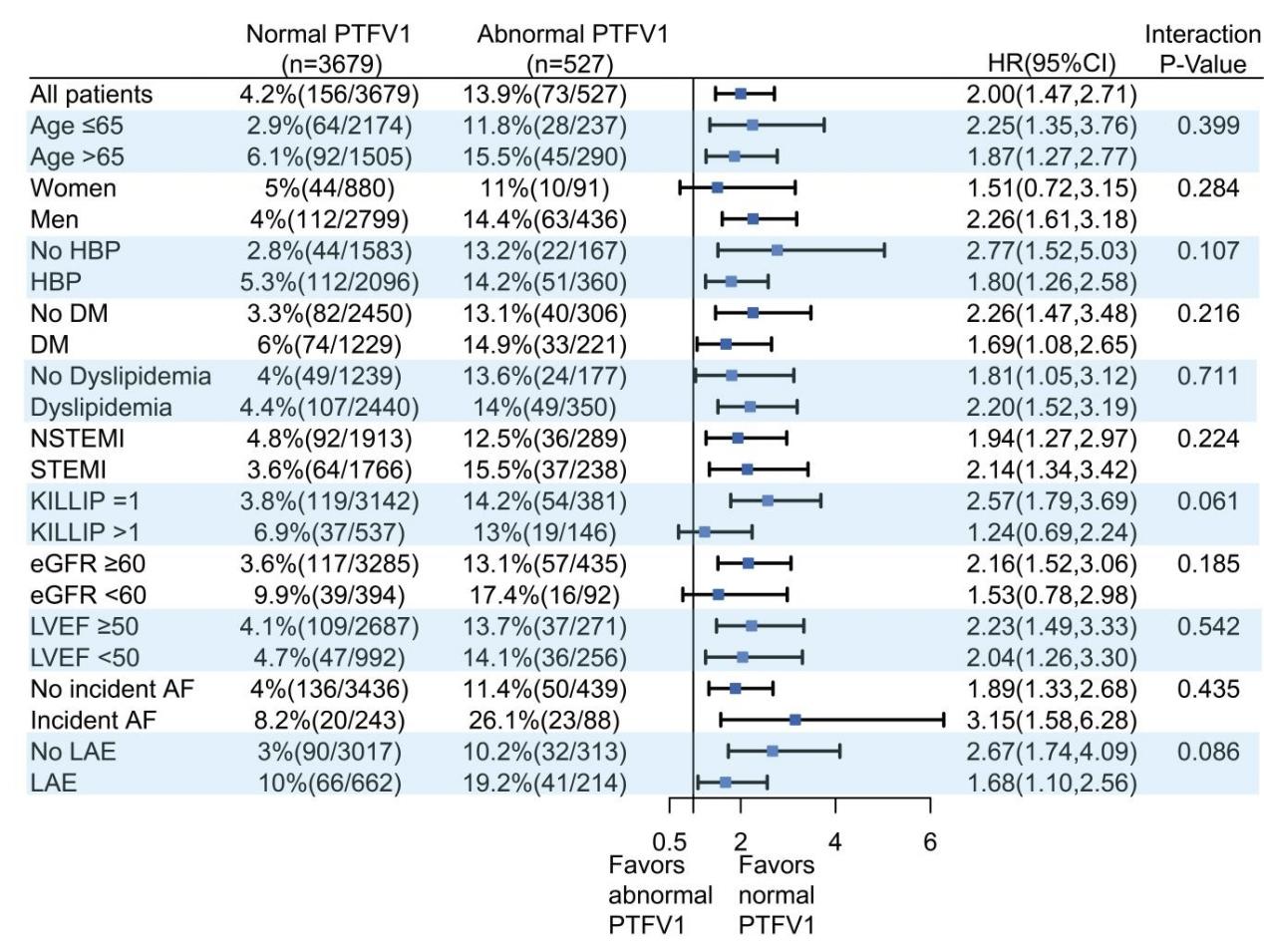
**

**FigS3**


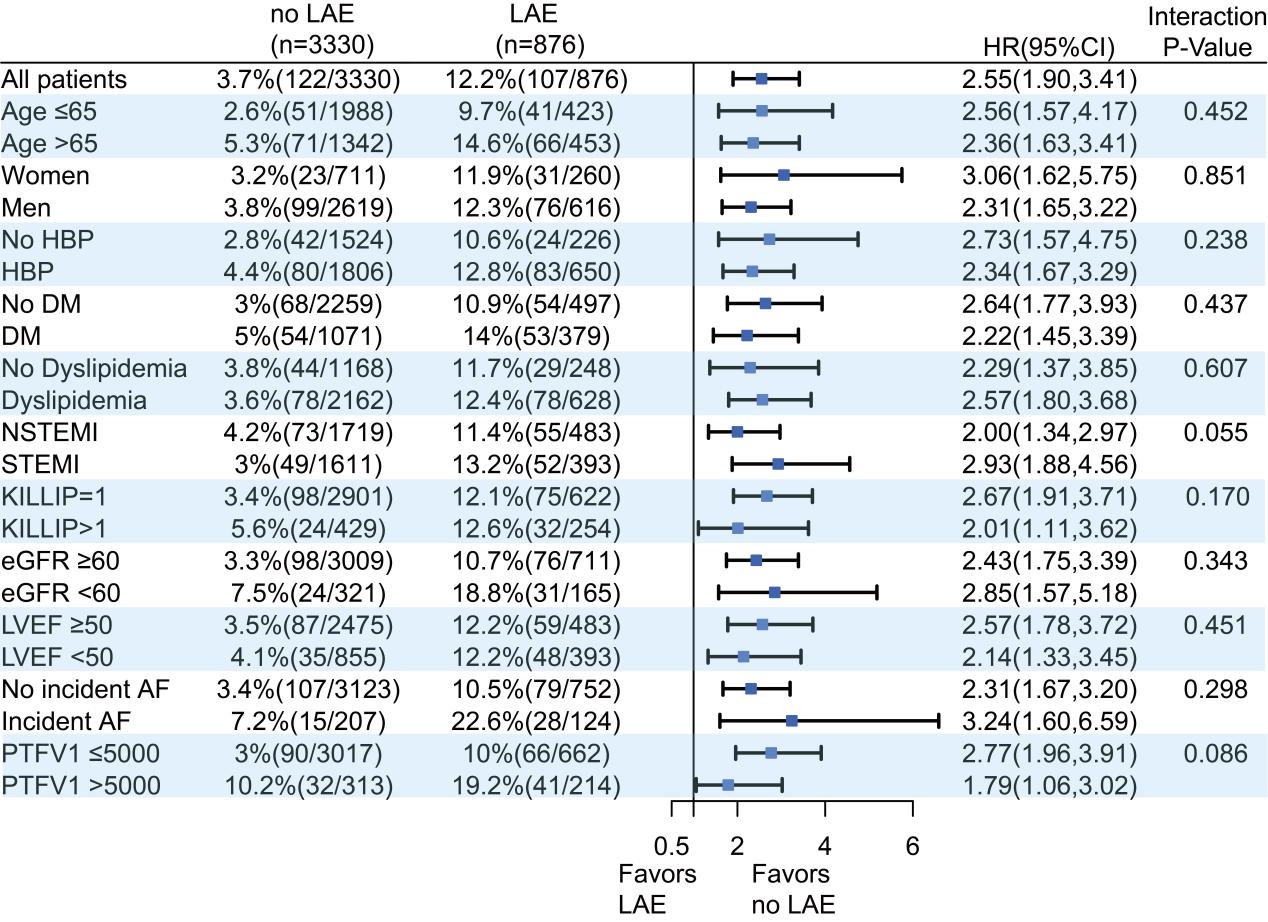

Supplement: Supplementary file 1 [file Table_1.DOCX]
